# Expression of Concern: Urban-rural disparities in institutional delivery among women in East Africa: A decomposition analysis

**DOI:** 10.1371/journal.pone.0328820

**Published:** 2025-07-23

**Authors:** The PLOS One Editors

The *PLOS One* Editors issue an Expression of Concern to alert readers that this article [1] was identified as one of a series of submissions for which we have concerns about authorship and adherence to the journal’s first and second publication criteria. We regret that these issues were not addressed prior to the article’s publication.
