# Supplementary material for: Urban-rural disparities in institutional delivery among women in East Africa: A decomposition analysis
Source: PLoS One. 2021 Jul 30;16(7):e0255094. doi: 10.1371/journal.pone.0255094 (PMC8323938; doi:10.1371/journal.pone.0255094)
Supplement: Attachment — Submitted filename: Authors Response2.docx [file pone.0255094.s001.docx]

**Authors’ responses to Reviewers and Editors Comments**

Dear Reviewers and editors;

We are very thankful for your invaluable comments. Here under are authors’ responses to the comments raised during review process.

**General comments on Journal Requirements**

**Comment 1:** 1. Please ensure that your manuscript meets PLOS ONE's style requirements, including those for file naming.

**Response 1:** As much as possible the authors were read the format and submission guidelines and hopefully it will fit with your recommendation.

**Comment 2:** We suggest you thoroughly copyedit your manuscript for language usage, spelling, and grammar.

**Response 2:** Melaku Yalew has revised the language part.

**Comment 3: d.** If you did not receive any funding for this study, please state: “The authors received no specific funding for this work.” Please include your amended statements within your cover letter; we will change the online submission form on your behalf.

**Responses 3:** The authors received no specific funding for this work

**Comment 4:** In your Data Availability statement, you have not specified where the minimal data set underlying the results described in your manuscript can be found. PLOS defines a study's minimal data set as the underlying data used to reach the conclusions drawn in the manuscript and any additional data required to replicate the reported study findings in their entirety. All PLOS journals require that the minimal data set be made fully available. For more information about our data policy, please see http://journals.plos.org/plosone/s/data-availability.

Upon re-submitting your revised manuscript, please upload your study’s minimal underlying data set as either Supporting Information files or to a stable, public repository and include the relevant URLs, DOIs, or accession numbers within your revised cover letter. For a list of acceptable repositories, please see http://journals.plos.org/plosone/s/data-availability#loc-recommended-repositories. Any potentially identifying patient information must be fully anonymized.

Important: If there are ethical or legal restrictions to sharing your data publicly, please explain these restrictions in detail. Please see our guidelines for more information on what we consider unacceptable restrictions to publicly sharing data: http://journals.plos.org/plosone/s/data-availability#loc-unacceptable-data-access-restrictions. Note that it is not acceptable for the authors to be the sole named individuals responsible for ensuring data access.

We will update your Data Availability statement to reflect the information you provide in your cover letter.

**Response 4:** The data underlying the study can be accessed after legal registration at www.measuredhs.com. After registration, interested researchers can log in at https://www.dhsprogram.com/data/dataset_admin/login_main.cfm and access the data as zipped files.

**Comment 5**: Your ethics statement should only appear in the Methods section of your manuscript. If your ethics statement is written in any section besides the Methods, please move it to the Methods section and delete it from any other section. Please ensure that your ethics statement is included in your manuscript, as the ethics statement entered into the online submission form will not be published alongside your manuscript.

**Response 5:** our ethics statement only appears in the Methods section and it is in our manuscript

**Comment 6**: Please include a copy of Table 5 which you refer to in your text in lines 380 and 707.

**Response 6**: table 5 is the currently of table 4

**Comment 7**: Please include your tables as part of your main manuscript and remove the individual files. Please note that supplementary tables (should remain/ be uploaded) as separate "supporting information" files.

**Response 7:** we have included our tables as part of main manuscript and removed the individual files.

**Comment 8:** Thank you for submitting the above manuscript to PLOS ONE. During our internal evaluation of the manuscript, we found significant text overlap between your submission and the following previously published works

**Response 8:** we have tried to address this comment by avoiding text overlap from our document as well as citing the citable references for our work.

**Reviewer 1**

Factors affecting the institutional delivery in LMIC is an important topic to explore. The authors used an interesting method to quantify the phenomenon. As authors stated the paper will be the first to use the Oaxaca-Blinder decomposition to examine the urban rural disparity in institutional delivery in selected east African countries

**Comment 1**: The paper is too long and most of information is not needed here or can be shortened

**Response 1:** we have tried to concise the document

Background:

**Comment 2:** The last paragraph of the background “page 12 lines 112-121”) can be moved to methods section

**Response 2:** we have deleted it from background

**Methods:**

**Comment 3:** Study population is just the women that have delivered in the past 5 years. **Question**: if a woman had delivered more than once in the past 5 years, please explain how are you exploring the information: are you considering all deliveries or just the last one?

**Response 3**: all births in the last five years were considered

**Results section**

**Comment 4**: Table 1 needs some statistical tests to these differences..

Table 2 needs some statistical tests and also there is a big problem for percentage of the “pregnancy-related deaths” (0.8%). The PRD is not affecting old women and most of deceases are women 60 years and above. You should recalculate this proportion based only on women of reproductive age (15-49years). This proportion will be much high that what the authors published. I think it will be more than 3%.

Please revise table 1 and table 2. The description in the manuscript is too confusing. I think on page 12, line 261 it is probably Table 2 and line 265 it is Table 1. Please review it again.

Table 1: Why not can provide the percentages instead of numbers? Too confusing.

What is the difference between table 1 and figure 1. I think they are providing the same information. If yes select only one of them

**Response 4:** Thank you very much, we have included chi-square and t-tests accordingly for categorical variables and continuous variables to show the presence of significant difference in place of residence**.** We have removed table 2 since the massage is similar with figure 1. Our study participants were 15 to 49 years women not 15 to 60 years and we haven’t any statement “pregnancy-related deaths” (0.8%)” in our document. Please you can check the result. Sorry for creating confusion we have corrected it by deleting table 2. The overlapping information reduced from table 1 and each has its own separate information.

**Comment 5** minor comments

**Response 5**: thank you, we have edited and deleted per comments.

**Comment 6:** Overall: the paper is too long and I will recommend the authors to shorten it by deleting or reducing several sections. For instance the last paragraph of the background is the same information that was described in the method. The description of the decomposition method is well known and can be shortened and authors can just provide references as needed. The results section can describe briefly the most important results and not provide too much information. At the end the readers are lost. Also the results sections should be shortened by describing results instead of providing some explanations in the results section with references in this section. All explanations and reference in the results section should be moved to Discussion section

**Response 6:** for this comments we have deleted parts from background, method and result accordingly without missing the flow of the manuscript.

**Reviewer #2: -**

**Abstract**

**Method**

**Comment 1: -**indicate that the study was in four east African countries

**Response 2:** we have mentioned that our analysis data was from “four East African countries”

**Comment 2**: covariate effect was dominate”; this contradicts with the result in the body

**Response 2:** we have corrected the body part that that the covariate effect is the dominant for the change in this study

**Conclusion**

**Comment 3: -**“regional inequality” is confusing;

-“facilitate antenatal care utilization” is not clear, which ANC care? Four or more? Be specific

-“plan number of children” is vague

**Response**: for clarity we have replaced regional inequality with residential inequalities,

- facilitate antenatal care utilization also replaced with to increase frequency of antenatal care utilization

- Plan number of children removed

**Introduction/background**

**Comment 4: Introduction/background**

**-**this section is an awkward and poorly written. You incoherently moved between different ideas; you begin with mortality, and then jumped to facility delivery and then back to mortality. Pls rewrite this section and ensure that the transitions between paragraphs are effective and clear. Also, you need to further substantiate it by adding points directly related with urban-rural disparity in facility delivery. Do not forget that you still follow certain chronological flows.

- in the “maternal mortality rate below 70”, change rate into ratio

-Lines 112-120: take this to method section

**Response 4:** we have critically considered the comment and we have rewritten the background section accordingly, please see the background for confirmation**.**

-we have changed rate into ratio

-we removed the method section from background

**Methods**

**Comment 5:** DHS involves women 15-49 and men 15 to 59 as well as children under five. You need to mention this in the method section through your analysis in this study has been restricted to only women. You need to also clarify the distinction between “study population” and “sample”. All women in the reproductive age group in the selected households are samples, not study populations, unlike what you have said. Pls avoid the confusion around these terms

**Response 5:** we have included in the method “DHS involves women 15-49 and men 15 to 59 as well as children under five. For the current study we used women 15-49 years”.

**-Our sample population: -** sample population was all reproductive-age women in each household at the enumeration area.

**Comment 6:** the “sampling and sample size” section is too vague to understand how and how many enumeration areas (ESs) and participants were enrolled into the study. It needs to be clearly expounded that the DHS follows a stratified two-stage cluster design, where, following stratification by both urban and regions/districts (you just mentioned that stratification was based solely on urbanization and that is false), eligible women were selected in two stages. In this section, you can explain that succinctly. How many EAs were sampled in the first stage? How many households were sampled in each EA? What was the household and individual response rates? What is primary sampling unit/cluster and SSU? Explain

**Response 6:**we have address these comments **as “**Urban-rural based stratified two stage cluster sampling was employed for each region. In the first stage clusters were selected randomly and in the second stage equal numbers of households were selected using systematic random sampling [3–6]. From Ethiopia, Kenya, Tanzania and Uganda 645, 1612, 608 and 697 clusters were sampled in the first stage respectively. For Ethiopia 28, Kenya 25, Tanzania 22 and Uganda 30 households per cluster were selected in the second stage. From these in Ethiopia 18008, Kenya 40,300, Tanzania 13,360 and Uganda 20,880 households were sampled. Overall the household response rate was 99% for Keya and 98% for Ethiopia, Tanzania and Uganda. From identified eligible women the response rate for women interview was 95% for Ethiopia and 97% for the rest countries. The current study incorporates 643 clusters and 10,547 births from Ethiopia [4], 1,593 clusters and 20,840 births from Kenya [5], 608 clusters and 10,175 births from Tanzania [3] and 696 clusters and 15,154 births from Uganda [6].”

**Comment 7**: place of residence is the stratifier variable, not an exposure/independent variable. It is a variable the inequality was measured by accounting for the influences of exposure variables

**Response 7:** we have corrected residence as “Stratification variable” since have learned the issue.

**Comment 8:** maternal age (age during the time of the surveys administrations) could not be an exposure variable, because it comes long after the outcome variable occurs. The word “cautious” is ambiguous in the “age at birth”

**Response 8: Thank you “Mr” Gebretsadik Shibre** for this critical comment make as to find solutions for the challenge and we compute the exact age of the mother at the birth of their child by subtraction of the year of birth b2 of the Child from the year of birth of the mother (v010). And reanalyze.

**Comment 9:** it is strange that you did not explain how these variables were selected, and no citation to prior similar works was made. You need to tell us that these variables show certain theoretical and or statistical relationship with the intended response variables.

**Response 9**: we have cited our sources that were used as a base for our classification.

**Comment 10:** Region is missing from being an exposure variable

**Response 10:** the reason we missed regions from exposure variables in our study is that regional classification is not similar to compare across study countries

**Comment 11**: Distance from health facility: if this variable was created based on the variable v467d, then that is incorrect. This variable reflects access problem at the time of the survey for a woman's medical help, and is not related with facility delivery.

**Response 11:** yes we used v467d, because we assume that access is one of the possible hindering factors for institutional delivery.

**Comment 12:** How was media exposure variable was created and produced? Explain that to allow replication for other studies as well as to improve transparency. This applies also to other variables.

**Response 12**: for clarity we put in the method section operational definition heading “women are considered as regularly exposed to media if they have at least one of the media (newspaper, radio or television) at least once a week otherwise considered as have no access”. For transparency we generate media exposure from three variables (frequency of reading newspaper or magazine (v157), frequency of listening to radio (v158) and frequency of watching television (v159) and three of them have three responses (not at all (0), less than once a week(1) and at least once a week (2)). Based on this information we generate media exposure as this do file we do

____________________________________________________________________

gen media_exposure=.

replace media_exposure=1 if v157==2|v158==2|v159==2

replace media_exposure=0 if media_exposure==.

label variable media_exposure "media exposure"

label define mediaexposure 0 "no accesses media" 1 "have accesses to media"

label values media_exposure mediaexposure

**Comment 13:** marital status variable was measured in DHS at the time of the survyes, and might not reflect marital status of the women before becoming pregnant, and therefore might not be an exposure variable for facility delivery; it has to come before or at least as the same time as the outcome variable to call it an exposure variable. Pls see this variable in the DHS guide for scrutiny.

**Response 13:** Really it is true some variation will be happen at time of birth and at the time of survey since marital status is time varying variable. However, the study design is cross-sectional which measure association but not able establish temporality. For this we acknowledged the limitations of the study.

**Analysis**

**Comment 14:** how did you assess the normality and Skewness of the variables? Explain that.

**Response 14:** we applied Skewness and kurtosis test with normal quantile plot to assess normality.

**Comment 15:** -you have described that the analyses were all ”population-weighted” and that “survey design” was taken into cognizant during analysis. This expression is very misleading for many reasons. First, the phrase “population-weighted” adds noting to understanding except that it brings confusion with it. You simply say you analysis is “weighted”. Second, you did not explain how your analysis should be weighted. Third, when we say, survey design is taken into account, we are saying that we are accounting for the disproportionate sampling (that we weight our analysis) and the stratification and clustering effect are taken into account to produce nationally representative estimates. So, the later expression, that is, the survey design is taken into account is enough and there is no any reason to repeat that analyses were weighted as far as you have mentioned that you have already mentioned that the complex nature of the data has been accounted for. Finally, when you say survey design was accounted for, I am not sure whether you have accounted for both stratification and clustering, and if you have missed one of them, then the estimated Standard errors were most likely be erroneous.

**Response 15:** we have used only **“**the survey design is taken into account”

**Comment 16**: it is not clear why you have also used the decomposition techniques suggested by powers et al after you have calculated BO decomposition? Both methods can be used of binary variables. But the problem with BO is that results could hugely differ depending on the reference category chosen during analysis, aka “identification problem”. So, with BO, YOU must do normalization to make the finding consistent with the chosen reference categories of categorical variables.

**Response 16:** so as to get detailed decomposition and normalized decomposition we used powers et al (Yun decomposition) and (nldecompose) for aggregate decomposition. However we missed the dummies and we have included in the revision.

**Comment 17: -** in the ethics section, please also mention that ethical procedures were the responsibility of the institutions that carried out the survey.

**Response 17: thank you,** we have mentioned it.

**Results**

**Comment 18: -**Since your outcome is facility delivery, just report that; reporting home deliveries adds only confusion

**Response18:** we have corrected with facility delivery percentage

**Comment 19:** the “delivery rate” is inappropriate usage as rate has a different meaning and does not go with delivery

**Response 19:** we have removed rates

**Comment 20**: the statement “however…, Table-1” is very vague and I cannot understand it

- The statement “Kenya has the highest 249 (35.83%) urban resident women

**Response 20**: sorry for making confusion, we have removed it but what we want to express is in the other country far most women reside in rural area.

**Comment 21:** lines 251-261: the texts here do not correspond to the content of table 1. Presented in table 1 is completely different from what the texts talks about. The texts about the differentials of urban and rural need to be presented well in terms of all the exposure variables so that we can see where these variables dominate, and this would then help us interpret the decomposition findings presented table 2 through table 4.

**Response 21**: here what we have tried is to compare the study areas by background characteristics with consideration of its importance to our discussion later.

**Comment 22**: Lines 262-265: it is the replication of table 2; pls avoid repetitions and redundancies

**Response 22:** really it is repetitions and we have removed

**Comment 23**: Lines 287-288: I think the contributions of the variables in the decomposition can be to either widen or narrow down the gap; this is the most interesting portion of the findings and hence requires further explanation in the discussion section. Why some variables contribute in terms of widening the gap, but not others?

**Response 23: we did**

**Comment 24:** lines 290-292: take this into the discussion

**Response 24:** we have moved it to discussion

**Comment 25**: -Lines 249-356: this paragraph actually lacks clear interpretations/presentations of findings. When you say that disparity could be reduced if urban and rural settings have similar levels of wealth, for instance, it can also be interpreted that disparity can be lowered if wealth in urban areas is reduced to a level in rural settings, which is not actually the case. So, you need to clearly present that when wealth in rural settings are increased to a level in urban areas, urban-rural disparities can be narrowed by certain amount. Also, make sure that in this text, you have not repeated what you have already presented in the Table.

**Response 25:** thank you based on the comment we have analyzed with dummies of the categorical variable to show the clear direction either to wide or narrow the gap.

**Comment 26:** Lines 357-359: in this small paragraph, you attempted to attach meaning to the observed contributions of the exposure variables. You are interpreting that variables with positive contribution like wealth tend to narrow down the disparity. However, how do these variables contribute this way? How do I know that these variables narrow, not widen the gap? I rather interpret the opposite way. I strongly recommend you to first document where these variables dominate (in urban or rural areas). For instance, if the richest and richer categories of wealth are predominantly found in the urban area, and if these categories have the capacity to increase the chance of giving birth in facilities, then surely, wealth tends to widen the disparity, rather than narrowing. Also, it is extremely important to show the contributions of each of the five categories of wealth, and of other categorical variables, and without doing that, you cannot interpret whether a variable contributes by widening or narrowing. At currently stands, wealth has 16% contribution to the disparity in Ethiopia, and we cannot know whether this contribution is to close the gap or not. My same comment applies to the other similar findings in the subsequent lines

**Response 26**: yes we have accepted the comment and addressed it as we have mentioned in response 25. We have also in showed that these factors are significantly different between urban and rural residences. Please see table 1.

**Discussion**

**Comment 27:** Lines 408-410: revise the vague statement

**Response 27**: we have tried to clarify by shortening sentences and selecting suitable words.

**Comment 28:** Lines 414-415: Even though there might not be a study on these countries together, there could be a number of studies that investigated facility delivery inequality in these countries, so unless you are 100% certain that there are no previous studies, you should rather avoid making such bold statement that there were no prio0r studies.

**Comment 29**: Lines 414-415: Even though there might not be a study on these countries together, there could be a number of studies that investigated facility delivery inequality in these countries, so unless you are 100% certain that there are no previous study, you should rather avoid making such bold statement that there were no prio0r studies.

**Response 29:** Thank you; yes it needs humble expression that is why we say to “our knowledge”.

**Comment 30:** Lines 420-427: you need to show that the variation has been explained by the factors whose contributions have been explored and presented in the tables in the result section. For instance, in these lines, you have mentioned that the urban-rural disparity has been partly explained by the disparity in formal education and at least one ANC (or the lack thereof). However, in the result sections, you did not show separately that such variables have contributed to the disparity. Also, the word “ignorance” has nothing to do with your finding and seems also unethical.

**Response 30:** Yes in the previous document we had missed it but in current document tables 3 and 4 answer this gap since we have reanalyzed per your comment. For the word ignorance we use uneducated mothers.

**Comment 31:** lines 433-435: it is important how the unequal distributions of endowments encourage urban women to attend to facility delivery. Which of these variables encourage urban women to deliver at facility, which variables discourage them? Explain the mechanism of actions of these variables to benefit women in urban and rural settings.

**Response 31:** we have mentioned variables and the possible mechanisms with reference

**Comment 32: -**lines 437-440: avoid the repetitions. How these variables widen the urban-rural gap?

**Response 32:** we have revised it to express additions rather than to repeat what already said

**Comment 33: -**lines 446-448: not clear, and needs some clarification to help the statement read well

**Response 33**: this expression was the combination of (table 1) covariate distribution differences between urban and rural women; and the facility delivery gap the will be narrowed if the gap in this covariate distribution increased in rural women (table 3).

**Comment 34:** lines 448-450: this interpretation is unclear; the evidence in the result section does not support the interpretations in these lines; how do we know that more frequent ANC visits could help urban women attend facility delivery in the absence of findings on the contributions of the specific categories ( that is 1-3 and >4= ANC visits) of the number of ANC visit?

**Response 34:** yes we have missed dummies and it was not directional. As we have addressed in response 25, this ambiguity will be solved in the current document.

**Comment 35:** for the subsequent paragraphs, there is a need to make a major revision and make sure that every piece of your interpretation is completely informed by your findings. Since the analysis is incomplete, this has seriously affected the interpretations of the findings in this section of the paper.

**Response 35**: similar with responses 25 and 34

**Comment 36**: -Also, it is good if you briefly elaborate on the policy implications of the study.

**Response 36:** we have tried to elaborate the policy implications for countries need covariate redistribution and behavioral interventions.

**Comment 37: -**it is not correct to blame the study being cross sectional as limitations of the study; causality cannot be made by a single study even with RCT, and more importantly, temporality is not the problem in the study at least for some of the variables like education and wealth. One important limitation in this is that in the BO decomposition, findings differ depending on the chosen reference categories of categorical variable, and hence requires further adjustment using “normalization” process. But, you need to overcome this problem in the study to get valid results.

**Response 37**: thank you for make us reconsider this limitation; however, our insight is that almost 16% of women at birth were less than 20 years. So they can change their education and wealth over time including with marriage or change in marriage. For identification problem we applied normalization.

**Comment 38**: I cannot assume that these findings are generalized to the east African community since these four countries do not represent the entire sub-region.

**Response 38: we have corrected with this “**The finding also generalized for similar countries as the data were representative and quality assured”

**Comment 39**: Your conclusion is not supported by the findings, pls see my comment in the result and discussion section. To higjlight on the contribution of wealth to the favor of the urban women, I should first see which categories of wealth ( poorest, poor, richest etc) dominate where ( urban or rural) and their respective contributions in the decompositions, because wealth is a categorical variable. Also, how do we specifically overcome the differential effect of the determinants to close the urban-rural gap in terms of use of facility delivery? That is an important dimension of your work and is better to further elaborate on how these can be challenged.

**Response 39:** we have addressed in result and discussion similar with responses 25 and 34

**Comment 40:** -the last statement of the conclusion has an incorrect word “strategist”

Response 40: corrected as “strategies”
