# Supplementary material for: Urban-rural disparities in institutional delivery among women in East Africa: A decomposition analysis
Source: PLoS One. 2021 Jul 30;16(7):e0255094. doi: 10.1371/journal.pone.0255094 (PMC8323938; doi:10.1371/journal.pone.0255094)
Supplement: Attachment — Submitted filename: Urban_rural_disparity_comments.docx [file pone.0255094.s002.docx]

**Urban-rural disparity in Institutional delivery in four Easter African countries**

I thank the authors for taking the time to revise their good paper in light with points suggested in my prior review. I read the revised version, and I would once again raise some points to help the paper fine-tuned.

**Background**

In every piece of your writing in this section, make sure you have touched issues for all the four countries under investigation. But as it currently stands, some paragraphs (e. g. the paragraph that comes after paragraphs which explain about maternal mortality burden) concentrated solely on situations of Ethiopia. Here, you mentioned about the coverage of skilled delivery in Ethiopia as of 2016 EDHS, what about coverage of the same indicator in the other study countries?

In this same paragraph, the statement “……..Even though women have accessed cost free 148 services, the proportion of institutional deliveries in Ethiopia was 26%” is a bit confusing; the physical services are in fact offered free of charge, but they are not available for free as women incur a lot of other costs to get the skilled maternal health care services. I suggest you revise it to read more clearly. The statement in the lines 151-152 is incomplete; it is a phrase.

The 79%(urban) vs.20% (rural) deliveries do not add up to 100%. Pls revise the figures. In this paragraph, the urban-rural scenario of skilled delivery was made for only Ethiopia and Kenya; you need to also do the same for the other countries, which you are going to assess their disparities. Without first establishing whether disparity exists between urban and rural in terms of your response variable, you could not logically go on to explain (through decomposition) the disparity. The last statement in this paragraph, that reads “These inconsistencies were 161 also well documented in different countries DHS and WHO reports” does provide only confusion, since no inconsistencies have been mentioned in that paragraph, which inconsistencies you are referring about? Instead, you have discussed similar findings about the existence of urban-rural inequality around institutional deliveries in developing nations including Ethiopia and Kenya.

Line 162 begins with “Other explanatory variables”; this has brought a huge confusion because in the preceding paragraph, you just were explaining about the existence of the urban-rural disparity, not about factors that drive the disparity. Mentioning that there is disparity is one thing; describing the factors that could underlie an observed disparity is another thing. I suggest you could give one paragraph for the latter case as the first case has already been done. The statement “However, these studies were lacking to determine 168 the observed differences in rates of health-facility deliveries between rural and urban women” requires more nuanced explanation to make it a strong reason for drawing attention to this topic in the first place; many attempts have already been made to show the difference between urban and rural settings in terms of institutional birth, but probably, what is lacking is that, this difference was not explained rigorously using the decomposition method you are using. I suggest you revise and substantiate this last paragraph of the background as readers want to see where the novelty of this study lies in the midst of a prolific number of similar articles on this same area. Finally, I look forward to seeing a reason that justify the restriction of the present study to just Easter Africa, while it is possible to do the same study in any other settings.

**Methods**

The first paragraph that details about the nature and purpose of DHS goes without citation.

The authors have substantially improved the section which details about sampling procedure for each country. But one thing that is conspicuously missing is that, you have said that stratification was done before selection of samples. However, it is not clear whether stratification was done on both place of residence and regions for all the four countries. On line 200, the statement that reads “Urban-rural based stratified two stage cluster sampling was employed for all regions” is too vague to understand; do you mean that stratification was done by region as well as place of residence for Ethiopia? Pls clarify that. Further, I am not sure whether the same applies for the other settings. I even wonder whether “regions” are available in the other countries, and other terms might have been used in the place of regions. I suggest to double check this. On the lines 200 and 201, you went on to say that “clusters were selected randomly”; nevertheless, I found this information not true since clusters are normally selected based on Probability Proportional to Size (PPS) approach, not randomly.

**Variables**

The way the response variable is measured varies by where deliveries took place; in Ethiopia, health extension workers can be counted as skilled attendant and by extension, delivery in health post can be treated as institutional delivery. So, it is good to briefly summarize how the response variable is measured in all the four settings.

I suggest that you change “stratification variable” into equity stratifier. The former confuses with the sampling procedure you mentioned prior to this section.

Some explanatory variables require further scrutiny. The variable “occupation” has many categories in DHS and I did not see all of them here. Cite a reason.

How can age of a woman **at birth** leads to institutional birth? Age at pregnancy could give more sense.

Why did you collapse wealth quintiles into three categories? Pls justify; shrinking the categories from 5 to 3 could result in loss of important information.

Any reason for using two methods as the same time; BO and multivariate decomposition (“mvdcmp” command) for decomposition?. I previously commented on the need to clarify whether authors have implemented “normalization” procedure when running BO decomposition as this method suffers from identification problem. Now, in the revised version, authors have provided the following response:

“so as to get detailed decomposition and normalized decomposition we used powers et al (Yun decomposition) and (nldecompose) for aggregate decomposition”. Here, why then you have used BO decomposition if everything has been done with the former method? Put another way, a BO decomposition that is not normalized results in a finding that varies by the type of reference group you chose in your analysis. For instance, if I were to re-analyze these same data and want to figure out the contribution of wealth, with the richest as referent group, then, surely, I will end up with a finding that is in no way comparable with what you have generated. This is the greater caveat of BO decomposition that could be minimized by undergoing normalization. If you have done this in this revised version, please indicate that. Plus, the “nldecompose” does not support all the three design elements I talked about somewhere in this recommendation.

On lines 239 and 240, in the statement “These included factors that are known to be associated with 240 institutional delivery and/or area of residency”, you should avoid the “and/or residency” as all of the presumed covariates/explanatory variables should be associated with facility delivery alone, not with place of residence; place of residence in this study serves only one purpose, serving as “equity stratifier”.

On the wealth index, you could add more information on which household possessions and durable materials were used to construct the index, and whether similar materials were used in all the four study areas. Furthermore, you need to back up the fact that the percentage of each of the four categories of wealth index deviates from 20% as shown in your result. But in the method section, you said that each category of wealth score accounts for 20%. Why this deviation?

The authors remain stubborn with their reason for ignoring region in their analysis. But, I would argue that, since they analyzed the data for each countries separately, it poses no problem if they include country specific variables in their analysis. Otherwise, omitting an important variable from for one or the other countries amounts to the so-called Omitted Variable Bias (OVB). Similarity of variables is must for pooled analysis, where an author needs to append one data beneath another to create one mega-data.

I see a problem in the “distance” variable; since you used this name to proxy access, make sure that the variable “distance” in this study refers exclusively to access. Plus, this variable was gathered at the time of the administration of the surveys, but institutional birth happened five years ago; how can these two variables be related as a presumed factor and outcome? Can this be highlighted as limitation? There are a few more variables that have such similar problems.

I thank the authors for clarifying the definitions of media exposure.

**Analysis**

It is not clear whether you have taken into account of all the three design elements (weighting, stratification, and clustering). It is extremely important for authors to be honest and explicitly state their analysis for other researchers to be able to replicate the analysis. DHS manual highly encourages researchers who are relying on DHS data to account their analysis for all of the three design elements.

I am pleased that the method has been improved since the first submission.

**Result**

I found it very difficult to judge whether a contribution is to narrowing or widening the urban-rural gap. I think it is worthwhile to briefly discuss in the method section on the techniques used to interpret the positive and negative contributions. For instance, how does “poor” contributed to widen the gap? since the largest proportion of poor was found in rural settings, you may be tempted to arguing that it widens the gap, but the same logic applies for rich category as the largest portion of this category is found among the urban settings. So, I may conclude that the rich as well widens the gap, but this time by helping women in urban areas to get most of the institutional delivery. Here, the difference between poor and rich’s contribution is the mechanism of action; the poor by preventing rural women from getting the service and the rich by promoting the urban women to get the service. So, I strongly recommend you to clearly state how one can easily interpret how and why an exposure variable contributes to widen or shrunken the observed urban-rural disparity. To this end, it would be useful to fit a logistic regression for urban and rural settings separately to see whether exposure variables have positive or negative effect on your outcome variable, and this would greatly assist the interpretations of their influence on the disparity. If the effect of the poor group is to really prevent women from getting their child delivered at facility, and if the largest percentage of the poor concentrates among the rural settings, then the poor could widen the gap. If the effect of the rich is to increase the uptake of institutional delivery and if the largest percentage of the rich is found among the urban settings, then, again, this widens the gap by stimulating women to get the service more than the poor rural women do. But, we do not this hidden interesting mechanism of action until you run a logistic regression to see the effect on the outcome variable of the exposure variables. Then, use the regression result to interpret the contributions as well as the mechanisms of action of the variables. Just take a look at the following statement from your analysis and see how unclear it is for readers, On line 484-485:

“poor wealth status (-36.55%) contributed for 485 widening of the gap and rich wealth status (70.40%) contributed for narrowing of this gap.” Why does the poor widen? Why does the rich helps to shrink the gap? See my explanation above to get this problem fixed.

Finally, what does unexplained portion of the decomposition imply? Does this correspond to discrimination? Pls explain it as it has a huge policy implication.

**Limitations**

The problems associated with the use of BO decomposition has not been duly explained in this section. The sheer use of BO decomposition is neither a problem nor a limitation by itself; pls describe a specific attribute of the method that you can consider as a weakness or limitation.

You have described that temporality remains to be a problem for the study. However, this limitation was not taken into account while interpreting your findings; if the temporality of the exposure variables were not known, why have you interpreted the findings as if they are like causes for institutional birth? When you say that wealth contributes to facility delivery, you are labeling at as a determinant factor. Pls pay attention to this discrepancy.

I highly recommend them to get the paper copyedited by someone highly proficient in English as poorly written manuscript is likely not to provide messages it intends to portray to readers. Also, I highly encourage them to edit the entire paper for some technical additions. Finally, I would like to thank the authors for their great work and for substantially improving the first version.

Regards,
