# Supplementary material for: Urban-rural disparities in institutional delivery among women in East Africa: A decomposition analysis
Source: PLoS One. 2021 Jul 30;16(7):e0255094. doi: 10.1371/journal.pone.0255094 (PMC8323938; doi:10.1371/journal.pone.0255094)
Supplement: Attachment — Submitted filename: Author response.docx [file pone.0255094.s003.docx]

**Author response to Reviewer**

**Dear Reviewer, Dr. Gebretsadik Shibrie**

We are very grateful for your constructive comments and suggestions. Here under are authors’ responses to the comments raised during second revision.

**Background**

**Comment 1:** In every piece of your writing in this section, make sure you have touched issues for all the four countries under investigation. But as it currently stands, some paragraphs (e. g. the paragraph that comes after paragraphs which explain about maternal mortality burden) concentrated solely on situations of Ethiopia. Here, you mentioned about the coverage of skilled delivery in Ethiopia as of 2016 EDHS, what about coverage of the same indicator in the other study countries?

**Response 1:** we have added the figures regarding to the rest three countries. (Kenya 61% [5], in Tanzania 63% [3] and in Uganda 74% [6])

**Comment 2:** In this same paragraph, the statement “……..Even though women have accessed cost free 148 services, the proportion of institutional deliveries in Ethiopia was 26%” is a bit confusing; the physical services are in fact offered free of charge, but they are not available for free as women incur a lot of other costs to get the skilled maternal health care services. I suggest you revise it to read more clearly. The statement in the lines 151-152 is incomplete; it is a phrase.

**Response 2:** we have rephrase like this, Even though women have accessed cost-free maternal and new-born care services, the proportion of institutional deliveries were in Ethiopia 26%......

**Comment 3:** The 79% (urban) vs.20% (rural) deliveries do not add up to 100%. Pls revise the figures.

**Response 3:** figure 20% is corrected as 21%.

**Comment 4:** In this paragraph, the urban-rural scenario of skilled delivery was made for only Ethiopia and Kenya; you need to also do the same for the other countries, which you are going to assess their disparities. Without first establishing whether disparity exists between urban and rural in terms of your response variable, you could not logically go on to explain (through decomposition) the disparity.

**Response 4;** for the rest of the countries we have added “In the case of Kenya urban institutional delivery was 82% and 48.5% in rural residents [5], in Tanzania urban 86.4% and rural 53.7% [3] and in Uganda it was reached 87.8% in urban and 69.5% in rural areas [6]. ”

**Comment 5:** The last statement in this paragraph, that reads “These inconsistencies were 161 also well documented in different countries DHS and WHO reports” does provide only confusion, since no inconsistencies have been mentioned in that paragraph, which inconsistencies you are referring about? Instead, you have discussed similar findings about the existence of urban-rural inequality around institutional deliveries in developing nations including Ethiopia and Kenya.

**Response 5:** inconsistency was replaced with “These urban-rural discrepancies”

**Comment 6:** Line 162 begins with “Other explanatory variables”; this has brought a huge confusion because in the preceding paragraph, you just were explaining about the existence of the urban-rural disparity, not about factors that drive the disparity. Mentioning that there is disparity is one thing; describing the factors that could underlie an observed disparity is another thing. I suggest you could give one paragraph for the latter case as the first case has already been done.

**Response 6**: Other explanatory variable was replaced with “From different literatures various explanatory variables were significantly linked with facility-based delivery; women education…..”

**Comment 7:** The statement “However, these studies were lacking to determine 168 the observed differences in rates of health-facility deliveries between rural and urban women” requires more nuanced explanation to make it a strong reason for drawing attention to this topic in the first place; many attempts have already been made to show the difference between urban and rural settings in terms of institutional birth, but probably, what is lacking is that, this difference was not explained rigorously using the decomposition method you are using.

**Response 7**: we have rephrase like this, “However, decomposition analysis method help to decomposing inequality in institutional deliveries in Urban-rural settings, this difference was not rigorously explained using the decomposition method.”

**Comment 8:** I suggest you revise and substantiate this last paragraph of the background as readers want to see where the novelty of this study lies in the midst of a prolific number of similar articles on this same area. Finally, I look forward to seeing a reason that justify the restriction of the present study to just Easter Africa, while it is possible to do the same study in any other settings.

**Response 8**: we have tried to rephrase as such “To develop effective strategies that minimize this urban-rural gap demands as such, a greater understanding of the underlying factors contributing to these disparities in institutional deliveries between urban and rural has paramount importance. So, this study aimed to identify the sources of variation in institutional delivery between the urban and rural areas in East Africa by using a decomposition analysis method.” Please see the manuscript.

**Methods**

**Comment 9:** The first paragraph that details about the nature and purpose of DHS goes without citation.

**Response 9**: thank you for reminding we have cited properly.

**Comment 10:** The authors have substantially improved the section which details about sampling procedure for each country. But one thing that is conspicuously missing is that, you have said that stratification was done before selection of samples. However, it is not clear whether stratification was done on both place of residence and regions for all the four countries. On line 200, the statement that reads “Urban-rural based stratified two stage cluster sampling was employed for all regions” is too vague to understand; do you mean that stratification was done by region as well as place of residence for Ethiopia? Pls clarify that.

**Response 10**: it was for all countries urban rural based but for Ethiopia both regional and residence based stratification was applied.

**Comment 11:** Further, I am not sure whether the same applies for the other settings. I even wonder whether “regions” are available in the other countries, and other terms might have been used in the place of regions. I suggest to double check this.

**Response 11:** yes you are right; in other countries regional classifications are geographical and are not similar with Ethiopia. The word regions to mean countries that were included in the study and we have corrected it.

**Comment 12:** On the lines 200 and 201, you went on to say that “clusters were selected randomly”; nevertheless, I found this information not true since clusters are normally selected based on Probability Proportional to Size (PPS) approach, not randomly.

**Response 12:** really we miss it and we have corrected per your comment and checking the approach from reports. Thank you!

**Variables**

**Comment 13:** The way the response variable is measured varies by where deliveries took place; in Ethiopia, health extension workers can be counted as skilled attendant and by extension, delivery in health post can be treated as institutional delivery. So, it is good to briefly summarize how the response variable is measured in all the four settings.

**Response 13:** For analysis, place of delivery was measured as a binary variable and was classified as “1” if a woman delivered in any healthcare facility and otherwise “0”. any healthcare facility is a facility minimum maternal cares and child care (bleeding control, vaccine and referral service). The name of minimum service providing facilities can be different health post in Ethiopia to dispensary Tanzania.

**Comment 14:** I suggest that you change “stratification variable” into equity stratifier. The former confuses with the sampling procedure you mentioned prior to this section.

**Response 14:** we have changed stratification variable to equity stratifier variable.

**Comment 15:** Some explanatory variables require further scrutiny. The variable “occupation” has many categories in DHS and I did not see all of them here. Cite a reason.

**Response 15**: our classification was for analysis amenity and interpretation.

**Comment 16**: How can age of a woman at birth leads to institutional birth? Age at pregnancy could give more sense.

**Response 16**: yes age at pregnancy is appropriate. However, this age is mathematically computed nearly age at birth minus one year, since there is no recorded pregnancy age for those recent gave births but for currently pregnant women. Moreover the variable is not significant in the current study in both collapsed and as the questionnaire form.

**Comment 17**: Why did you collapse wealth quintiles into three categories? Pls justify; shrinking the categories from 5 to 3 could result in loss of important information.

**Response 17**: Since in different literatures both classifications were used to make our manuscript concise we have reported the three classification approach.

**Comment 18**: Any reason for using two methods as the same time; BO and multivariate decomposition (“mvdcmp” command) for decomposition?. I previously commented on the need to clarify whether authors have implemented “normalization” procedure when running BO decomposition as this method suffers from identification problem. Now, in the revised version, authors have provided the following response:

“so as to get detailed decomposition and normalized decomposition we used powers et al (Yun decomposition) and (nldecompose) for aggregate decomposition”. Here, why then you have used BO decomposition if everything has been done with the former method? Put another way, a BO decomposition that is not normalized results in a finding that varies by the type of reference group you chose in your analysis. For instance, if I were to re-analyze these same data and want to figure out the contribution of wealth, with the richest as referent group, then, surely, I will end up with a finding that is in no way comparable with what you have generated. This is the greater caveat of BO decomposition that could be minimized by undergoing normalization. If you have done this in this revised version, please indicate that. Plus, the “nldecompose” does not support all the three design elements I talked about somewhere in this recommendation.

**Response 18**: nldecompose with different weighting provide results of Oaxaca, Blinder, Reimer, and Cotton that is why we prefer it for aggregate decomposition.

Authors prefer multivariate decomposition for detailed decomposition analysis and interpretation suit as both methods used in normalized detailed decomposition. As we have read ” mvdcmp is primarily intended for use in nonlinear decomposition and is based on recent contributions, which include convenient methods to handle path dependency (Yun 2004), computing asymptotic standard errors (Yun 2005a), and overcoming the identification problem associated with the choice of a reference category when dummy variables are included among the predictors (Yun 2005b, 2008).

To be transparent and for any comments suit if we are expected to learn our command become this one as a sample

mvdcmp residence1, normal (wealth_index1 wealth_index2 wealth_index3| distance1 distance2 | husb_occup1 husb_occup2 husb_occup3 husb_occup4| media_exposure1 media_exposure2| hus_educ1 hus_educ2 hus_educ3| ANCF1 ANCF2 | No_live_child1 No_live_child2 No_live_child3 ): logit p_delivery wealth_index1 wealth_index3 distance1 husb_occup2 husb_occup3 husb_occup4 media_exposure2 hus_educ2 hus_educ3 hus_educ4 ANCF2 No_live_child1 No_live_child3 age [pw=wt]

\# In the introduction if the Oaxaca decomposition make confusion we replace with decomposition method.

**Comment 19**: On lines 239 and 240, in the statement “These included factors that are known to be associated with 240 institutional delivery and/or area of residency”, you should avoid the “and/or residency” as all of the presumed covariates/explanatory variables should be associated with facility delivery alone, not with place of residence; place of residence in this study serves only one purpose, serving as “equity stratifier”.

**Response 19**: thank you, but in this comment was addressed based on your previous comment.

**Comment 20**: On the wealth index, you could add more information on which household possessions and durable materials were used to construct the index, and whether similar materials were used in all the four study areas. Furthermore, you need to back up the fact that the percentage of each of the four categories of wealth index deviates from 20% as shown in your result. But in the method section, you said that each category of wealth score accounts for 20%. Why this deviation?

**Response** 20: this classification is originated from DHS guide. So, all countries use similar classification. “Households are given scores based on the number and kinds of consumer goods they own, ranging from a television to a bicycle or car, and housing characteristics such as source of drinking water, toilet facilities, and flooring materials. These scores are derived using principal component analysis. National wealth quintiles are compiled by assigning the household score to each usual (de jure) household member, ranking each person in the household population by her or his score, and then dividing the distribution into five equal categories, each comprising 20% of the population.” (Uganda,Tanzania , Ethiopia and Kenya DHS) as well as DHS guide.

As we have mentioned in methods section for the current analysis we merge poorest and poorer to poor and richer and richest to rich and middle as it is so each three category account for 40%, 40% and 20%.

**Comment 21**: The authors remain stubborn with their reason for ignoring region in their analysis. But, I would argue that, since they analyzed the data for each countries separately, it poses no problem if they include country specific variables in their analysis. Otherwise, omitting an important variable from for one or the other countries amounts to the so-called Omitted Variable Bias (OVB). Similarity of variables is must for pooled analysis, where an author needs to append one data beneath another to create one mega-data.

**Response 21**: yes it could be another limitation but we have no other reason other than we have mentioned in first review.

**Comment 22**: I see a problem in the “distance” variable; since you used this name to proxy access, make sure that the variable “distance” in this study refers exclusively to access. Plus, this variable was gathered at the time of the administration of the surveys, but institutional birth happened five years ago; how can these two variables be related as a presumed factor and outcome? Can this be highlighted as limitation? There are a few more variables that have such similar problems.

**Response 22**: yes you are right it is one of the limitations of the cross-sectional study design that suffer from temporal relationship. As you said some individuals status during interview may be different from at a time of child birth and it is not for all and we couldn’t avoid in cross-sectional study. Moreover, if see years lived in place of residence (v104) more than 90% were resided 5 and more years and as you Know the expansion of health facility is not significantly high in this period. It was mentioned as a limitation in the first manuscript.

**Analysis**

**Comment 23**: It is not clear whether you have taken into account of all the three design elements (weighting, stratification, and clustering). It is extremely important for authors to be honest and explicitly state their analysis for other researchers to be able to replicate the analysis. DHS manual highly encourages researchers who are relying on DHS data to account their analysis for all of the three design elements.

**Response 23**: to be honest the only think we account is probability weighting. The reason is we cannot found a command that can account the three survey natures. We have tried to see the effect of

svyset v021 [pweight=wt], strata(v022) vce(linearized) singleunit(missing) on mvdcmp but it has no effect. But only weight has shown difference from unweight outcome.

**Result**

**Comment 24** I found it very difficult to judge whether a contribution is to narrowing or widening the urban-rural gap. I think it is worthwhile to briefly discuss in the method section on the techniques used to interpret the positive and negative contributions. For instance, how does “poor” contributed to widen the gap? since the largest proportion of poor was found in rural settings, you may be tempted to arguing that it widens the gap, but the same logic applies for rich category as the largest portion of this category is found among the urban settings. So, I may conclude that the rich as well widens the gap, but this time by helping women in urban areas to get most of the institutional delivery. Here, the difference between poor and rich’s contribution is the mechanism of action; the poor by preventing rural women from getting the service and the rich by promoting the urban women to get the service. So, I strongly recommend you to clearly state how one can easily interpret how and why an exposure variable contributes to widen or shrunken the observed urban-rural disparity. To this end, it would be useful to fit a logistic regression for urban and rural settings separately to see whether exposure variables have positive or negative effect on your outcome variable, and this would greatly assist the interpretations of their influence on the disparity. If the effect of the poor group is to really prevent women from getting their child delivered at facility, and if the largest percentage of the poor concentrates among the rural settings, then the poor could widen the gap. If the effect of the rich is to increase the uptake of institutional delivery and if the largest percentage of the rich is found among the urban settings, then, again, this widens the gap by stimulating women to get the service more than the poor rural women do. But, we do not this hidden interesting mechanism of action until you run a logistic regression to see the effect on the outcome variable of the exposure variables. Then, use the regression result to interpret the contributions as well as the mechanisms of action of the variables. Just take a look at the following statement from your analysis and see how unclear it is for readers, On line 484-485:

“poor wealth status (-36.55%) contributed for 485 widening of the gap and rich wealth status (70.40%) contributed for narrowing of this gap.” Why does the poor widen? Why the rich does helps to shrink the gap? See my explanation above to get this problem fixed.

**Response 24**: for the fear of the length of the manuscript and the reviewer 1 comment most of the methodology should be cited not fully discussed. We prefer to only cite the materials. To clarify our result, the negative coefficients indicate the less sensitivity of the higher group for that particular factor like poor wealth that means if rural poor women became as equal as urban women wealth the institutional delivery in rural women become higher than urban women. The protective effects of poor wealth in home delivery are not as strong for urban as they are for rural. We have tried to discuss the meaning and implication of the wealth in the discussion part and we showed urban rural significant wealth difference in the descriptive analysis.

The implication of positive coefficient is if that factor become equalised between lower group and higher group (rural and urban) the outcome (institutional delivery difference in high and lower group become narrow). The implication of negative coefficient is that the higher groups are less sensitive to that factor and if the lower groups get the chance of equality with their factor of difference the change in outcome may become greater than the higher group.

Regarding to logistic regression we have fitted it before we conduct multivariate decomposition to select the factors and visualise their effect. Why we miss in the result is to minimise size of the manuscript. For a simple demonstration the effect of wealth in urban and rural separately supports our argument.

**Comment 25**: Finally, what does unexplained portion of the decomposition imply? Does this correspond to discrimination? Pls explain it as it has a huge policy implication.

**Response 25**: Unexplained (coefficient) effect is the effect of the factor rather than the distribution of factors or behavioural changes and that need quality of the factors rather than the distribution of the factors. We have put the policy implication of unexplained portion immediately before the limitation.

**Limitations**

**Comment 26**: The problems associated with the use of BO decomposition has not been duly explained in this section. The sheer use of BO decomposition is neither a problem nor a limitation by itself; pls describe a specific attribute of the method that you can consider as a weakness or limitation.

**Response 26**: Although Oaxaca method has limitations like, Ordering of variables problem, Index problem, Observations matching problem and Choice of sample weights, we have applied Oaxaca extension **mvdcmp** approach to hand this. That is why preferred to reserve from mentioning limitations.

**Comment 27**: You have described that temporality remains to be a problem for the study. However, this limitation was not taken into account while interpreting your findings; if the temporality of the exposure variables were not known, why have you interpreted the findings as if they are like causes for institutional birth? When you say that wealth contributes to facility delivery, you are labelling at as a determinant factor. Pls pay attention to this discrepancy.

**Response 27**: The authors consider, understanding the cross-sectional nature of the study and appreciating its limitation like temporality problem in the limitation section will portray this gaps.

**Comment 28**: I highly recommend them to get the paper copyedited by someone highly proficient in English as poorly written manuscript is likely not to provide messages it intends to portray to readers. Also, I highly encourage them to edit the entire paper for some technical additions.

**Response 28**: we have tried to show our paper for language and grammar editing with language expert in our university

**Comment 29**: Finally, I would like to thank the authors for their great work and for substantially improving the first version.

**Response 29**: Really I am speechless to thank reviewer (Gebretsadik Shibrie) for your time dedication, constructive comments and helping in extensive improvement of the manuscript. Given comments are not only for current manuscript improvement but also increasing our overall understanding of the analysis and research. We may not satisfy you in addressing all issues even we have tried, but we really learnt from what we have done in future as well.
